# Stress reduction and psychological therapy for IBS: a scoping review

**DOI:** 10.3389/fgstr.2024.1342888

**Published:** 2024-05-02

**Authors:** Anjali J. T. Fernandes, Anna L. Farrell, Sara V. Naveh, Subhankar Chakraborty

**Affiliations:** ^1^ The Ohio State University College of Medicine, Columbus, OH, United States; ^2^ Department of Gastroenterology, Hepatology and Nutrition, The Ohio State University, Columbus, OH, United States

**Keywords:** irritable bowel syndrome, psychotherapy, stress reduction, quality of life, CBT

## Abstract

**Introduction:**

Irritable Bowel Syndrome (IBS) is a highly prevalent functional gastrointestinal disease that is commonly associated with psychological comorbidities and maladaptive thought patterns. Previous studies report psychological therapies such as cognitive behavioral therapy (CBT) and gut-directed therapy (GDP) improve IBS symptom management and quality of life. This review seeks to understand the effectiveness of various psychotherapies across delivery methods for patients with irritable bowel syndrome.

**Methods:**

A scoping literature review of PubMed articles highlighting psychological and stress reduction treatments for IBS was conducted. 120 studies were included in the title and abstract screening. 32 studies were selected for full text review. Primary and secondary research studies that investigated the benefit of psychological therapies focusing on stress reduction and cognitive therapies for patients with gastrointestinal condition’s symptom relief met inclusion criteria for the review. 12 studies were selected for inclusion.

**Results:**

All 12 reviewed studies reported statistically significant improvements in IBS symptoms with psychological therapies. 8 studies also addressed quality of life and reported statistically significant improvement in intervention groups. 3 studies demonstrated persistent improvement after 12 months. 2 studies compared different types of psychotherapies and reported improvements compared to control groups but no significant differences between psychotherapies. 6 studies that compared face to face therapy with minimal contact or telephone therapy showed no difference in clinical outcomes.

**Discussion:**

Psychological therapies demonstrate reported statistically significant improvements in IBS symptoms and patient quality of life with no reported statistically significant difference across forms of healthcare delivery. Most improvements reportedly persist long-term. Further research with a broader demographic base is needed to assess the economic costs of psychological therapies and their implications for underserved communities.

## Introduction

Irritable bowel syndrome (IBS) is an extremely common diagnosis in the United States affecting between 25 to 45 million people ranging from adolescence into old age (1). Symptoms of this functional diagnosis may include diarrhea, constipation, fecal incontinence, chronic functional abdominal pain, or distension (2). Annual direct costs in the United States have reportedly cost up to $1.35 billion annually (3). In addition to expenses from emergency department (ED) visits, routine appointments, and generalized healthcare, patients must also pay for the laboratory and imaging diagnostic workup and extensive time lost from work (4). In 2003, medical expenses reportedly averaged $619 annually to patients, likely only heightened by inflation (3).

There is currently an assortment of medications and procedural interventions used to treat IBS. In their article, Lacy et al. explain that IBS is commonly managed multimodally with diet and lifestyle changes, psychotherapy, and pharmacotherapy. The authors argue the potential of further research into these alternative therapies and functional treatments for IBS (5). The usual care of IBS patients revolves around symptom relief with few studies focused on alternative methods like cognitive behavioral therapy (CBT). Research exploring the therapeutic benefits of psychotherapy and stress reduction for health extends beyond gastroenterology into other fields such as cardiology (6), rheumatology (7), hospice care, and family medicine (8). While individual studies demonstrate temporary relief in regard to quality of life and mental health in patients with IBS, there is little conclusive, updated, holistic review of the value of CBT and gut-directed psychotherapy holds for patients regarding their IBS and psychological symptoms.

Patients with IBS commonly have psychological comorbidities, although a causal connection between these two domains has yet to be illustrated. A systematic review performed by Fond et al. in 2014 found that patients with IBS had reportedly statistically significant higher levels of anxiety and depression than those without the diagnosis (9). This demonstrates a clear need for research investigating the relationship of psychological conditions with IBS and the dual benefits in their treatment. A desire for less physically invasive IBS treatments has led to the rise of psychotherapies in practice such as CBT (10). Individual studies have indicated the benefit of psychological therapy for psychological symptoms as well as abdominal symptoms as well. This review seeks to understand the effectiveness of various psychotherapies relative to usual IBS care across in-person, virtual, and hybrid delivery methods for patients with irritable bowel syndrome.

## Methods

### Search criteria strategy and selection

A scoping literature review was conducted on psychological and stress reduction treatments of gastrointestinal conditions. Several PubMed searches were performed, all of which had filters of full-text English language primary and secondary articles published after 2010. The first PubMed search of “Irritable Bowel Syndrome/therapy” AND (“self-care” OR “hope”) yielded 44 results. A second PubMed search of “Stress, Psychological/prevention and control”[MAJR] AND “gastrointestinal” yielded 35 results. A final third search “Stress, Psychological/prevention and control” AND “gastrointestinal” contributed 66 results. 32 studies yielded in the three searches were duplicates. A total of 113 studies were included in the initial title and abstract screening. During full-text review, references were reviewed and seven articles were included in our review contributing to a final count of 120 publications for screening.

**Figure 1:**
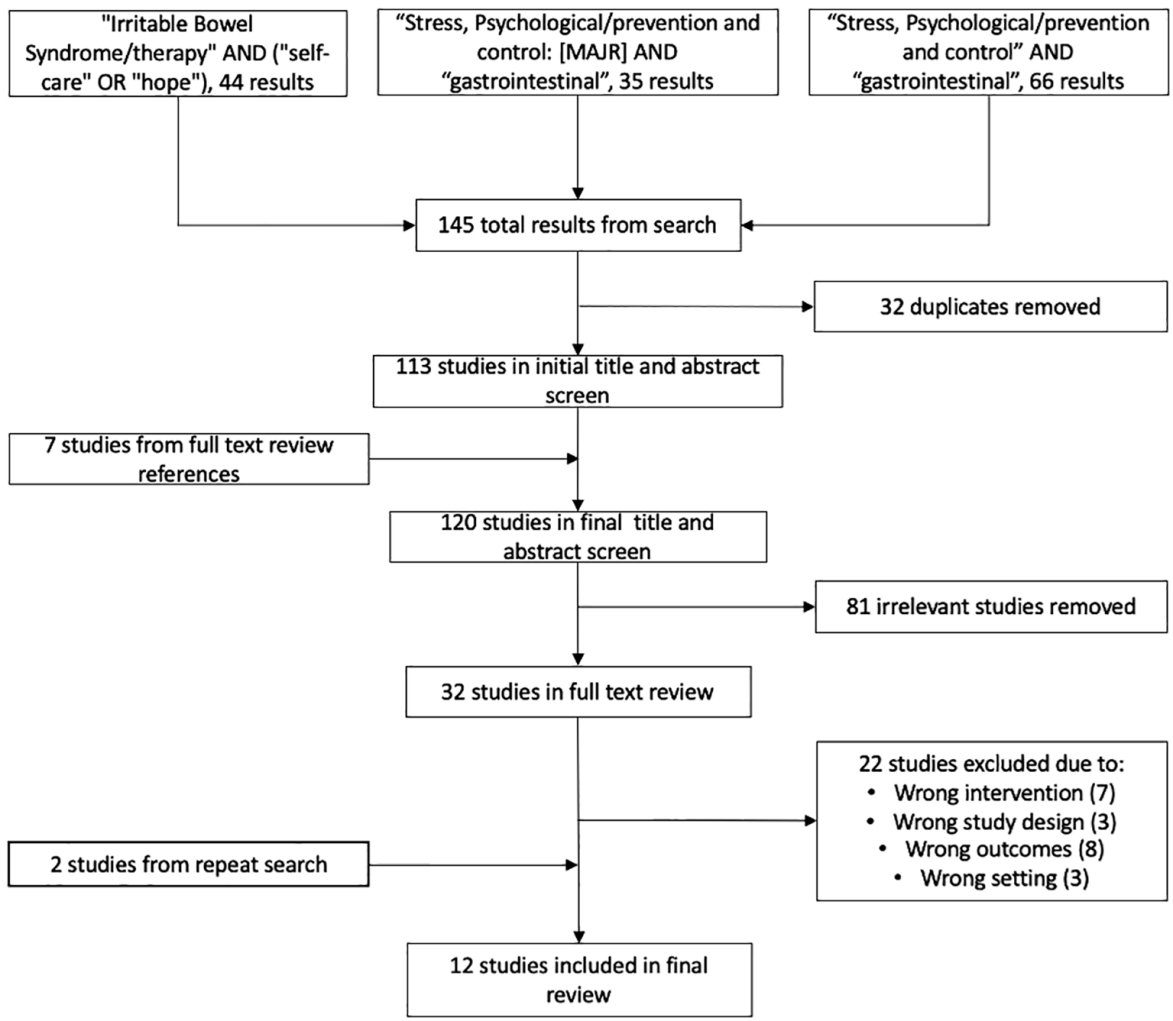
Flow diagram for assessment and selection of studies included in scoping review.

Title and abstract screening was performed and 32 articles were approved by three of the authors for further evaluation with a full-text review. Inclusion criteria was met by English literature articles written since 2000 that investigated the use of psychotherapies for the relief of gastrointestinal symptoms. During full-text screening, primary and secondary research studies that investigated the benefit of psychological therapies for patients with gastrointestinal condition’s symptom relief met inclusion criteria for the review. Articles that did not meet all elements of inclusion criteria were excluded from review. While some publications addressed self-management or patient education programs, if a predominant focus of these programs was on stress reduction and cognitive therapies met inclusion criteria if clinical gastrointestinal outcomes were primary measures. Authors independently conducted their own full-text review and met to achieve a consensus. Two additional studies were included following reference reviews of selected articles. At the conclusion of the literature review, 12 articles were included (**Figure 1**). Because all 12 articles successfully addressed our research question through peer reviewed randomized control trials and met inclusion criteria, we determined that they were of appropriate quality for this review.

### Data extraction and organization

Information was extracted from the articles included in the review and organized into tables for comparison. Demographic information including study location, study design, mean patient age, gender breakdown, and reported statistically significant differences in demographics was extracted (**Table 1**). Further information regarding the study methodology was extracted including the following: study sample sizes, duration of follow-up, percentage in control and intervention arms, treatment details, outcomes, and treatment delivery method (**Tables 2**, **3**).

**Table 1:** Demographics of included randomized control trials.

Author, Year	Location	Mean age in years (standard deviation)	% Males	% Females	Reported statistically significant differences at baseline between groups
Blanchard, 2007 (11)	New York, USA	49.8 (12.7)	16.1	83.9	None reported
Heitkemper, 2004 (12)	Washington, USA	33 (30)	0	100	Predominant bowel pattern (p =.03): - constipation-prone (UC*, 14%; Brief, 15%; Comprehensive, 18%); - diarrhea-prone (UC, 7%; Brief, 31%; Comprehensive, 10%) Severe abdominal pain (30% in UC, 25% in Brief, 50% in Comprehensive)
Jarrett, 2009 (13)	Washington, USA	44 (14)	13.7	86.3	None reported
Kennedy, 2006 (14)	London, UK	33.8 (8.6)	18.3	81.7	None reported
Lackner, 2008 (15)	New York, USA	46.6 (16.7)	14	86	None reported
Lackner, 2018 (10)	New York, USA	41.4 (14.8)	19.7	80.3	None reported
Nes, 2013 (16)	Oslo, Norway	Not reported	Not reported	Not reported	None reported
Oerlemans, 2011 (17)	Utrecht, The Netherlands	Control: 40.6 (15.5) Intervention: 35.9 (11.7)	Control: 13.1 Intervention: 8.1	Control: 76.9 Intervention: 91.9	Control group had significantly more diarrhea symptoms

UC *, usual care.

**Table 2:** Randomized control trial article interventions and measured outcomes.

Author, Year	Follow up	Sample Size	Intervention group (%)	Control group (%)	Intervention	Control	Outcomes measured	Intervention administrator
Blanchard, 2007 (11)	2 weeks, 3 months	210	79	21	Cognitive therapy: 10 weekly 90-minute sessions Psychoeducational support: 10 weekly 90-minute sessions primarily educational	Symptom monitoring (switched to CT after post-treatment assessment)	Primary: individual IBS symptoms, McGill Pain Questionnaire, overall functioning/side effects Secondary: physician and patient global IBS ratings and rating of change in sense of well being	Clinical psychologist or trained post-doc fellows
Heitkemper, 2004 (12)	Immediate (Comprehensive) or 9 weeks (Brief), 6 months, 12 months	144	Comprehensive: 36 Brief: 34	33	Comprehensive: 8-session multicomponent program Brief: Single session multicomponent program	Usual Care	Improved symptoms Psychological distress Quality of life Indicators of stress-related hormones.	Psychiatry Nurse Practitioner
Jarrett, 2009 (13)	3 months, 6 months, 12 months	188	67	33	Comprehensive self-management in person: 9 one-hour sessions, 13 weeks Telephone sessions: 3 in person, 6 over the phone, 13 weeks	Usual care	Primary: SSS, IBS-QoL Secondary: Psychological distress, cognitive beliefs, work productivity and activity, quality control blinding safety measures	Psychiatry Nurse Practitioner
Kennedy, 2006 (14)	3 months, 6 months, 12 months	149	48	52	CBT+ Mebeverine therapy	Mebeverine therapy only	Primary: SSS Secondary: WASA, illness perception, beliefs about medicine, adherence to medication, client service receipt inventory, CSFBD, behavior scale for IBS	Trained nurse
Lackner, 2008 (15)	12 months	75	64	36	S-CBT: 10 weekly, 60 min sessions MC-CBT: self-study CBT materials; 4 60-minute clinic visits	Wait list control	Primary: CGI-I, adequate relief from pain/symptoms Secondary: psychological distress, SSS, IBS-QoL, CSS	S-CBT: therapist MC-CBT: therapist
Lackner, 2018 (10)	3 months, 6 months	436	67	33	S-CBT: 10 weekly, 60 min sessions MC-CBT: home-study materials, 4 sessions of CBT	IBS Education (4 sessions)	IBS symptom improvement: CGI-I Secondary index: SSS Quality of care: CSS	S-CBT: therapist MC-CBT: therapist
Nes, 2013 (16)	3 months	76	50	50	4 weeks CBT with electronic diaries, individualized written situational feedback on PDA	Usual Care	Primary: PCS Secondary: IBS-QoL, CSFBD, abdominal pain, GI symptoms	Psychologist
Oerlemans, 2011 (17)	4 weeks, 3 months	76	49	51	Standard care+ 4 weeks CBT with electronic diaries, individualized written situational feedback on PDA	Usual Care	Written symptom questionnaires CSFBD IBS-QoL PCS Abdominal pain	Psychologist

Interventions: CT, cognitive therapy; CBT, cognitive behavioral therapy; MC-CBT, Home-based/minimal therapist contact CBT; PDA, personal digital assistants; S-CBT, Standard CBT.

Outcome metrics utilized: CGI-I, Clinical global impressions improvement; CSFBD, Cognitive Scale for Functional Bowel Disorders; CSS, Client satisfaction scale; IBS-QoL, IBS quality of life questionnaire; PCS, Pain catastrophizing scale; SSS, Symptom Severity Scale; WASA, hospital anxiety and depression, work, and social adjustment.

**Table 3:** Systematic review article descriptions and measured outcomes.

Author, Year	Location	Studies Included	Intervention	Outcomes measured	Intervention administrator
Ahl, 2013 (18)	Adelaide, Australia	9 RCTs	7: CBT vs. control 1: Self-help guidebook	IBS symptoms Psychological coping Quality of Life Primary care visits	Self-administered with no or minimal therapist contact
Black et al, 2020 (19)	Leeds, UK; Texas and North Carolina, USA; Ontario, Canada	41 RCTs	CBT or gut directed hypnotherapy	IBS symptoms	CBT: self-administered, face to face, group, or minimal contact (telephone) Gut directed hypnotherapist
Ford, 2019 (20)	Leeds, UK; Florida, Arizona, and Texas USA; Ontario, Canada	53 RCTs	17: antidepressants vs. placebo 35: psychological therapy vs. usual management 1: psychological therapy and antidepressants vs. placebo	IBS symptoms	Varied throughout studies
Zijdenbos, 2009 (21)	Utrecht, Netherlands	25 studies	17: CBT vs control/placebo 10: Stress relief/relaxation therapy 3: interpersonal psychotherapy	IBS symptoms Abdominal pain Quality of Life	Varied throughout studies

## Results

### Description of studies

The initial search yielded 145 total results, 12 of which were included in the final review. 4 of these studies were systematic reviews of previous randomized control trials (RCTs) comparing psychological interventions to either a usual care or placebo group (18–21). Psychological interventions included cognitive behavioral therapy (CBT), intrapersonal psychotherapy, dynamic psychotherapy, and stress reduction or relaxation therapy. As one study investigating psychopharmacology (tricyclic antidepressants and SSRIs) did so relative to psychotherapy and care as usual, it was deemed to meet inclusion criteria (20). 4 studies compared psychological interventions to usual care provided by the physician (12, 13, 16, 17). Alternate controls employed in the reviewed studies include patients receiving symptom management treatment (11), on a wait-list (15), IBS symptom education (10), or mebeverine medical therapy (14). Of the 8 RCTs included in our review, 6 used CBT as the primary psychological intervention in comparison to a control (10, 11, 14–17). Two of the 6 RCTs using CBT studies compared minimal contact CBT or over the telephone CBT to in person CBT (10, 15). Meanwhile the other two RCTs performed by Jarrett 2009 and Heitkemper 2004 utilized comprehensive multicomponent therapy that included education, diet counseling, relaxation, stress management, and CBT (12, 13).

### Study outcomes

#### IBS symptoms

All 12 studies demonstrated reportedly statistically significant improvement in IBS symptoms with psychosocial interventions compared to the control groups. Across studies, clinical outcomes were measured through patient self-reports including individual symptom questionnaires addressing symptoms such as pain, abdominal tenderness, flatulence, bloating, diarrhea, and constipation or through the standardized Symptom Severity Score (SSS) or Clinical Global Impressions (CGI). In studies utilizing the SSS, statistically significant improvements were reported with the use of CBT in three studies (13, 14, 21) and non-significant improvements in 1 study (10). In Lackner et al., 2018, improvements were significant in CGI (10). Six studies demonstrated reported statistically improvements in pain or abdominal tenderness (11, 15–17, 20, 21) for intervention groups compared to control. Seven studies demonstrated reported statistically significant improvement in all symptoms (11–13, 15, 18–20). One study found reported statistically significant improvements in symptom control with psychoeducation (11). Many studies additionally demonstrated non-significant improvements in other symptom areas (10–12, 17). Ultimately, all studied intervention groups demonstrated clinically significant improvement in symptoms relative to studied control groups.

#### Quality of life

**Figure 2:**
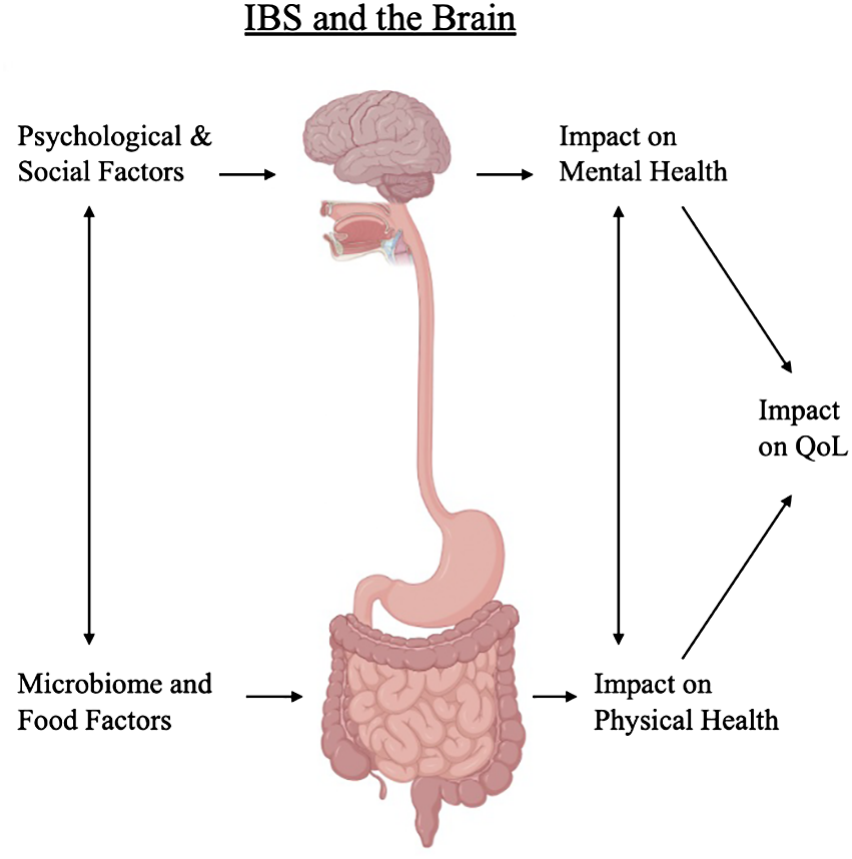
Relationship between the psychological, social, and environmental factors on development of IBS and quality of life.

Of the 12 studies reviewed, 8 assessed Quality of Life (QOL) in intervention groups compared to control or usual care (11–13, 15–18, 21). QOL measurements included the IBS-QOL questionnaire, psychological distress questionnaires, e-diaries, patient self-reported catastrophizing thoughts, the Cognitive Scale for Functional Bowel Disorders (CS-FBD) the Work and Social Adjustment Scale (WASA), and patient satisfaction surveys. All intervention groups demonstrated reported statistically significant improvements in their respective measured QOL outcomes. Psychotherapy effectively improved QOL in all 8 studies (11–13, 15–18, 21). Catastrophizing thoughts decreased significantly in 3 studies (12, 16, 17) with persistently lowered levels over 3 months in one study (16). One study found fewer primary care visits at long term follow up with reduced health care costs (18). One study additionally found improvements in the WASA with CBT interventions (14). Jarrett et al. similarly demonstrated reported statistically significant long term improvement in QOL for routine CBT and minimal contact CBT. This article also reported that secondary outcomes of psychological distress, cognitive beliefs, work productivity, and activity worsened for the control group at long term follow up (13). Nearly all studies demonstrated persistent improvements in overall QOL for intervention groups (**Figure 2**).

#### Duration of improvements

Multiple studies evaluated outcomes long-term. 3 studies observed patients at a 3 month follow-up with significant improvements in catastrophizing thoughts (16, 17), QOL, fewer primary care visits (18), pain (11, 16–18), bloating (11), and flatulence (11). One study continued to assess outcomes and demonstrated persistent reported statistically significant SSS score reductions at 6 months of follow up (10). 4 studies addressed follow up at 3, 6, and 12 months (12–15). 3 of these studies demonstrated persistent improvements at 12 months in the intervention group (12, 13, 15). Of these 3 studies, one compared comprehensive and brief interventions with reported statistically significant improvements persisting for 12 months only in the comprehensive intervention group (12). The fourth study with a 12 month follow-up demonstrated waning improvements after 6 months follow-up in symptom severity and QOL (14).

#### Type of psychosocial therapy

Two studies compared different types of psychological therapies to each other as well as against a control. One study included CBT, relaxation therapy, stress management, dynamic psychotherapy, multi-component therapy, and hypnotherapy (20). All forms of psychotherapy demonstrated improvement in global IBS symptoms greater than the control group. Interestingly, there was minimal difference between psychotherapies in terms of outcome. The second study included CBT, interpersonal psychotherapy, and relaxation therapy (21). Each type of psychotherapy was assessed in terms of overall symptom relief, IBS symptoms, abdominal pain, and quality of life improvements. All groups demonstrated improvement in all outcomes compared to the control group. There was no significant difference between groups in terms of which outcome was most affected or significance of improvement.

#### Delivery form of psychosocial therapy

Six studies compared in-person therapies to minimal contact or phone therapy. All 6 studies demonstrated no statistically significant differences in outcomes between minimal contact or phone therapy with in-person therapy (10, 13, 15, 16, 18, 19). However, Ahl et al., 2013 reported that self-directed therapy without a therapist had worse outcomes compared to minimal contact therapy (18). Two studies compared psychosocial interventions to education or symptom monitoring (10, 11). One study found that there were more responders to CBT when compared to simple IBS education (10). The other study found that psychoeducation and CBT both led to significant improvements in nearly all outcomes except for global improvement, which was better for CBT (11).

## Discussion

This study reviewed articles that examined the effects of psychotherapies on patient reported IBS symptoms. This review examines multiple forms of psychotherapies such as CBT, stress and relaxation therapy, interpersonal therapy, and comprehensive educational and lifestyle therapies. Furthermore, we compare the duration of treatment effects after psychological therapies to control outcomes. We address both symptom improvement, such as abdominal pain, flatulence, bloating, diarrhea, and constipation, and quality of life outcomes as described by psychological distress, working ability, and catastrophizing thoughts.

This is one of few reviews of psychotherapies on patient-reported IBS symptoms since the onset of the COVID-19 pandemic to the authors’ knowledge. We felt it crucial to include reviews conducted since the onset of the COVID-19 pandemic due to the increased utility of telepsychology and virtual delivery methods after the pandemic. Furthermore, the pandemic catalyzed an increase of psychosocial stressors that contribute to increased IBS flares. Thus, its analysis of the influence of psychotherapeutic care delivery has on gastrointestinal clinical outcomes is highly relevant. The results demonstrating comparable efficacy of self-administered, virtual, and minimal therapist contact psychotherapy services relative to routine therapy following the clinical limitations imposed COVID-19 pandemic has great potential in expanding healthcare access given the recent rise in telehealth services and advocacy for increased telehealth insurance coverage (22, 23). While telehealth has greatly enhanced healthcare access, it exposed a digital divide for those with disabilities (24) or limited skill with digital devices, access to broadband internet, and technology (25). Despite the reported shrinking of this gap, it persists especially for women, Black (26), and low-income patients (27). Studies intending to elucidate psychiatric telehealth utilization often meet with difficulty when extrapolating the manner with which patients engage with health services (28). Therefore, the studies reviewing psychiatric teletherapy in this article (13, 16, 17) uniquely testify to clinical benefit for patients with IBS. Although challenges in telemental health provision exist (29), the application of new Current Procedural Terminology codes for therapeutic monitoring of CBT patients will encourage adoption with better reimbursement (30).

One other review written since the COVID-19 pandemic was included in this study and similarly reviewed psychological interventions in IBS management (19). Black et al. primarily focused on symptomatic outcomes of IBS following a variety of psychological therapies. This study is superior to Black et al. because it not only addresses symptomatic clinical outcomes, but also the quality-of-life improvements from psychological therapies in IBS treatment. We also included interventions that addressed crucial elements of IBS management such as diet, stress and relaxation, and education. This study was reviewed and discussed by 3 authors to ensure high quality and relevance.

The studies selected for review demonstrated that psychological therapy significantly improved IBS patient symptoms and quality of life. 6 of the 8 RCTs utilized standardized scales such as the SSS or CGI which allowed results to be compared equally across studies. Similarly, quality of life was measured by patient satisfaction surveys or standardized scales such as the IBS-QOL and CSFBD. Psychological therapies such as CBT, interpersonal therapy, and stress and relaxation therapies had significant improvements in clinical outcomes and quality of life. In the studies that compared different types of psychotherapies, there was negligible difference in outcomes between types of psychotherapies. The included studies demonstrated improvements with psychotherapies compared to usual care, education, and symptom management. Furthermore, studies that measured outcomes at long term follow up found that symptom and quality of life improvements persisted longer in intervention groups.

### Future directions and limitations

Most studies included in the review’s subjects were primarily female and white, belonging to the middle-upper socioeconomic status. While IBS has been noted predominantly in white women (31), these studies overlook the potential benefits for males, racial minorities, and lower socioeconomic classes. It has been noted that racial minority IBS patients receive less specialist consultations and are subjected to more aggressive and costly procedural workups than typically employed in routine care (32, 33). While studies included in this review commonly excluded comorbid conditions and disabilities to minimize confounding, this neglected the study of psychotherapies’ influence in these communities’ gastrointestinal complaints. The included studies did not report psychological comorbidities in their patients which could confound the relationship of the therapies to changes in actual outcomes or improvement in mental health.

Several studies did not account for patient income status, a demographic particularly salient when monitoring IBS symptoms, as low-income patients often experience increased stress which may aggravate symptoms. Heitkemper et al. took this into account and noted that patients lost to follow-up in their study were less likely to be married or partnered and less likely to have an annual family income greater than $40,000 (p values of 0.12 and 0.13 respectively) (12). This finding urges the scientific community to take these demographics into consideration more consistently when evaluating barriers to health maintenance.

While all studies evaluated gastrointestinal clinical outcomes, few measured the clinical utility and economic outcomes of psychological IBS treatment (10, 18). Ahl et al., 2013’s systematic review included one study that reported a 60% reduction in primary care visits relative to the control group after receiving psychotherapeutic treatment (18, 34). None of the papers reviewed in our study measured income differences, healthcare utilization, or costs to patients and the system. Furthermore, the different QOL metrics used across studies varied in their ability to account for economic stress, healthcare access, and social stressors. This made it difficult to compare QOL outcomes between studies as well as assess the holistic impact of psychological IBS treatments. Again, the absence of recording patient socioeconomic status in studies clouds analysis as worsened stress could inflame IBS symptoms leading to increased healthcare utilization and higher health costs.

Our study did have limitations due to the available literature on the subject and lack of standardization in evaluating interventions. The broad range of psychological interventions and metrics used to in the reviewed studies created difficulty in comparison and mislead interpretations. Although some reviewed studies included self-reported catastrophic thoughts, psychological distress questionnaires, and CS-FBD as measures of QOL (14, 17), these are the very thought patterns psychological interventions target to improve patient QOL. Therefore, their use as a metric for QOL indirectly assumes the association under inquiry, that psychological interventions improve the QOL and symptom burden that patients experience.

As only 12 publications met inclusion criteria, this topic demands more rigorous study. Future research into this area should focus on understudied populations, diversifying subjects involved in the studies and accounting for patients’ racial, disability, and economic status especially. While the use of psychological therapies has been associated with decreased primary care visits and overall symptom improvement, the cost of intensive psychological therapy programs is significant. Many of these programs required a long duration of treatment (> 2 months) with external requirements for symptom monitoring, dietary changes, and homework following sessions. This review suggests that greater patient choice in modality and regimen of psychotherapy does not compromise the symptomatic relief they may experience from their IBS treatment. By evaluating economic outcomes and clinical utility, research may guide interventions and policies that aim to expand healthcare access and improve IBS patients’ lives.

## Conclusion

This literature review demonstrated significant symptomatic and quality of life improvement in IBS with the use of psychological therapies. CBT was the most studied psychological therapy and demonstrated long term effectiveness in symptom management and quality of life. Furthermore, there was little difference in results for in person therapies compared to telephone, internet, or minimal contact therapies. Very few studies met inclusion criteria indicating that this topic demands continued study. Further research into this area should focus on economic costs of psychological therapies, their application in underserved populations, and diversity of patient groups.

## Author contributions

AJTF: Conceptualization, Data curation, Formal Analysis, Investigation, Project administration, Visualization, Writing – original draft, Writing – review & editing. ALF: Conceptualization, Data curation, Formal Analysis, Investigation, Methodology, Project administration, Resources, Supervision, Writing – original draft, Writing – review & editing. SN: Data curation, Formal Analysis, Investigation, Software, Visualization, Writing – original draft. SC: Conceptualization, Investigation, Project administration, Resources, Supervision, Visualization, Writing – review & editing.

## References

[1] IBS facts and statistics international foundation for gastrointestinal disorders. Available online at: https://aboutibs.org/what-is-ibs/facts-about-ibs/.

[2] Simrén M Palsson OS Heymen S Bajor A Törnblom H Whitehead WE. Fecal incontinence in irritable bowel syndrome: Prevalence and associated factors in Swedish and American patients. Neurogastroenterol Motil. (2016) 29. doi: 10.1111/nmo.12919 PMC5276715 27581702

[3] Inadomi JM Fennerty MB Bjorkman D. Systematic review: the economic impact of irritable bowel syndrome. Aliment Pharmacol Ther. (2003) 18:671–82. doi: 10.1046/j.1365-2036.2003.t01-1-01736.x 14510740

[4] Konturek PC Brzozowski T Konturek SJ. Stress and the gut: pathophysiology, clinical consequences, diagnostic approach and treatment options. J Physiol Pharmacol. (2011) 62:591–9. 22314561

[5] Lacy BE Pimentel M Brenner DM Chey WD Keefer LA Long MD. ACG clinical guideline: management of irritable bowel syndrome. Am J Gastroenterol. (2021) 116:17–44. doi: 10.14309/ajg.0000000000001036 33315591

[6] Linden W Phillips MJ Leclerc J. Psychological treatment of cardiac patients: a meta-analysis. Eur Heart J. (2007) 28:2972–84. doi: 10.1093/eurheartj/ehm504 17984133

[7] Greco CM Rudy TE Manzi S. Effects of a stress-reduction program on psychological function, pain, and physical function of systemic lupus erythematosus patients: a randomized controlled trial. Arthritis Rheumatol. (2004) 51:625–34. doi: 10.1002/art.20533 15334437

[8] Hunter J. The role of psychotherapy in a general hospital. Am J Psychother. (2020) 73:117–8. doi: 10.1176/appi.psychotherapy.20200029 33317329

[9] Fond G Loundou A Hamdani N Boukouaci W Dargel A Oliveira J. Anxiety and depression comorbidities in irritable bowel syndrome (IBS): a systematic review and meta-analysis. Eur Arch Psychiatry Clin Neurosci. (2014) 264:651–60. doi: 10.1007/s00406-014-0502-z 24705634

[10] Lackner JM Jaccard J Keefer L Brenner DM Firth RS Gudleski GD. Improvement in gastrointestinal symptoms after cognitive behavior therapy for refractory irritable bowel syndrome. Gastroenterology. (2018) 155:47–57. doi: 10.1053/j.gastro.2018.03.063 29702118 PMC6035059

[11] Blanchard EB Lackner JM Sanders K Krasner S Keefer L Payne A. A controlled evaluation of group cognitive therapy in the treatment of irritable bowel syndrome. Behav Res Ther. (2007) 45:633–48. doi: 10.1016/j.brat.2006.07.003 16979581

[12] Heitkemper MM Jarrett ME Levy RL Cain KC Burr RL Feld A. Self-management for women with irritable bowel syndrome. Clin Gastroenterol Hepatol. (2004) 2:585–96. doi: 10.1016/S1542-3565(04)00242-3 15224283

[13] Jarrett ME Cain KC Burr RL Hertig VL Rosen SN Heitkemper MM. Comprehensive self-management for irritable bowel syndrome: randomized trial of in-person vs. combined in-person and telephone sessions. Am J Gastroenterol. (2009) 104:3004–14. doi: 10.1038/ajg.2009.479 PMC2804069 19690523

[14] Kennedy TM Chalder T McCrone P Darnley S Knapp M Jones RH. Cognitive behavioural therapy in addition to antispasmodic therapy for irritable bowel syndrome in primary care: randomised controlled trial. Health Technol Assess. (2006) 10:iii–iv,ix-x,1-67. doi: 10.3310/hta10190 16729918

[15] Lackner JM Jaccard J Krasner SS Katz LA Gudleski GD Holroyd K. Self-administered cognitive behavior therapy for moderate to severe irritable bowel syndrome: clinical efficacy, tolerability, feasibility. Clin Gastroenterol Hepatol. (2008) 6:899–906. doi: 10.1016/j.cgh.2008.03.004 18524691 PMC2630498

[16] Nes AA Eide H Kristjánsdóttir ÓChecktae van Dulmen S. Web-based, self-management enhancing interventions with e-diaries and personalized feedback for persons with chronic illness: a tale of three studies. Patient Educ Couns. (2013) 93:451–8. doi: 10.1016/j.pec.2013.01.022 23433735

[17] Oerlemans S van Cranenburgh O Herremans PJ Spreeuwenberg P van Dulmen S. Intervening on cognitions and behavior in irritable bowel syndrome: A feasibility trial using PDAs. J Psychosom Res. (2011) 70:267–77. doi: 10.1016/j.jpsychores.2010.09.018 21334498

[18] Ahl A Mikocka-Walus A Gordon A Andrews JM. Are self-administered or minimal therapist contact psychotherapies an effective treatment for irritable bowel syndrome (IBS): a systematic review. J Psychosom Res. (2013) 75:113–20. doi: 10.1016/j.jpsychores.2013.04.008 23915766

[19] Black CJ Thakur ER Houghton LA Quigley EMM Moayyedi P Ford AC. Efficacy of psychological therapies for irritable bowel syndrome: systematic review and network meta-analysis. Gut. (2020) 69:1441–51. doi: 10.1136/gutjnl-2020-321191 32276950

[20] Ford AC Lacy BE Harris LA Quigley EMM Moayyedi P. Effect of antidepressants and psychological therapies in irritable bowel syndrome: an updated systematic review and meta-analysis. Am J Gastroenterol. (2019) 114:21–39. doi: 10.1038/s41395-018-0222-5 30177784

[21] Zijdenbos IL de Wit NJ van der Heijden GJ Rubin G Quartero AO. Psychological treatments for the management of irritable bowel syndrome. Cochrane Database Syst Rev. (2009) 1:CD006442. doi: 10.1002/14651858 19160286

[22] S.3791 - Access to Prescription Digital Therapeutics Act of 2022. USA: Senate Finance Committee (2022). 117 Sess. Available at: https://www.congress.gov/bill/117th-congress/senate-bill/3791

[23] Salsabili M Tesell M Alcusky M Greenwood BC Huang D Lenz K. Prescription digital therapeutics: Applying Medicaid experience to value assessment and formulary management. J Manag Care Spec Pharm. (2023) 29:685–91. doi: 10.18553/jmcp.2023.29.6.685 PMC10387922 37276040

[24] Valdez RS Rogers CC Claypool H Trieshmann L Frye O Wellbeloved-Stone C. Ensuring full participation of people with disabilities in an era of telehealth. J Am Med Inform Assoc. (2021) 28:389–92. doi: 10.1093/jamia/ocaa297 PMC7717308 33325524

[25] Crawford A Serhal E. Digital health equity and COVID-19: the innovation curve cannot reinforce the social gradient of health. J Med Internet Res. (2020) 22:e19361. doi: 10.2196/19361 32452816 PMC7268667

[26] Williams DR Priest N Anderson NB. Understanding associations among race, socioeconomic status, and health: Patterns and prospects. Health Psychol. (2016) 35:407–11. doi: 10.1037/hea0000242 PMC4817358 27018733

[27] Saeed SA Masters RM. Disparities in health care and the digital divide. Curr Psychiatry Rep. (2021) 23:61. doi: 10.1007/s11920-021-01274-4 34297202 PMC8300069

[28] Torous J Michalak EE O’Brien HL. Digital health and engagement-looking behind the measures and methods. JAMA Netw Open. (2020) 3:e2010918. doi: 10.1001/jamanetworkopen.2020.10918 32678446

[29] Rapfogel N. The Behavioral Health Care Affordability Problem. USA: Center for American Progress (2022).

[30] Ekekezie O Hartstein GL Torous J. Expanding mental health care access-remote therapeutic monitoring for cognitive behavioral therapy. JAMA Health Forum. (2023) 4:e232954. doi: 10.1001/jamahealthforum.2023.2954 37713208

[31] Andrews EB Eaton SC Hollis KA Hopkins JS Ameen V Hamm LR. Prevalence and demographics of irritable bowel syndrome: results from a large web-based survey. Aliment Pharmacol Ther. (2005) 22:935–42. doi: 10.1111/j.1365-2036.2005.02671.x 16268967

[32] Ahuja A. Minority patients with irritable bowel syndrome receive more gastroenterology-related procedures than white counterparts: nuances in disparities research. Gastroenterology. (2022) 162:1354–5. doi: 10.1053/j.gastro.2021.10.023 34688709

[33] Sasegbon A Vasant DH. Understanding racial disparities in the care of patients with irritable bowel syndrome: The need for a unified approach. Neurogastroenterol Motil. (2021) 33:e14152. doi: 10.1111/nmo.14152 33835634

[34] Robinson A Lee V Kennedy A Middleton L Rogers A Thompson DG. A randomised controlled trial of self-help interventions in patients with a primary care diagnosis of irritable bowel syndrome. Gut. (2006) 55:643–8. doi: 10.1136/gut.2004.062901 PMC1856107 16099784

